# Snus use and risk of schizophrenia and non-affective psychosis

**DOI:** 10.1016/j.drugalcdep.2016.04.035

**Published:** 2016-07-01

**Authors:** Marcus R. Munafò, Sara Larsson Lönn, Jan Sundquist, Kristina Sundquist, Kenneth Kendler

**Affiliations:** a MRC Integrative Epidemiology Unit at the University of Bristol, Bristol, United Kingdom; b UK Centre for Tobacco and Alcohol Studies, School of Experimental Psychology, University of Bristol, Bristol, United Kingdom; c Center for Primary Health Care Research, Lund University, Malmö, Sweden; d Virginia Institute for Psychiatric and Behavioral Genetics, Department of Psychiatry, Virginia Commonwealth University School of Medicine, Richmond, VA, USA

**Keywords:** Tobacco, Snus, Schizophrenia, Non-affective psychosis

## Abstract

**Background:**

Recent studies suggest a possible causal role for smoking in schizophrenia and psychosis. Most studies have focused on cigarette smoking, the most common form of tobacco use, but other forms of tobacco exist, including smokeless products such as Swedish snuff (or “snus”).

**Methods:**

We explored whether snus use is associated with schizophrenia and non-affective psychotic illness in a large Swedish registry data set. The majority of participants were aged 18 or 19 at the time of assessment.

**Results:**

We observed a positive association between snus use and odds of schizophrenia in all analyses, but the magnitude of the association was small and the confidence interval wide, consistent with no association (fully adjusted HR 1.03, 95% 0.70–1.54). A similar pattern was observed for non-affective psychosis, but the magnitude of the association was somewhat greater and the confidence intervals narrower, so that these analyses provided stronger statistical evidence for this association (fully adjusted HR 1.22, 95% CI, 1.00–1.48).

**Conclusions:**

Our results therefore provide modest evidence for an association between snus use and risk for non-affective psychosis. This is consistent with emerging evidence from a range of studies and methodologies that tobacco use may be a risk factor for psychotic illness. However, our results provide some evidence against the hypothesis that it is the burnt products of cigarette smoke that are psychotogenic.

## Highlights

• Emerging evidence suggests tobacco use may be a risk factor for psychotic illness.
• We observed an association between smokeless tobacco use and non-affective psychosis.
• This provides further evidence that tobacco may increase risk of psychotic illness.
• Any causal effect of tobacco use may not be due to combustion products of tobacco.

## 1 Introduction

Tobacco use prevalence is considerably higher among psychiatric patients, including people with schizophrenia, compared with the general population (de Leon and Diaz, 2005). For the most part, it has been assumed that this comorbidity reflects, at least in part, self-medication on the part of individuals with schizophrenia, to remediate either the symptoms of the disease, or the side-effects of antipsychotic medication. The possibility that the association may reflect a causal effect of tobacco use on schizophrenia risk has not received widespread consideration, despite the fact that tobacco use typically predates the onset of psychotic symptoms. If smoking is indeed a causal risk factor for schizophrenia, then this has important implications for public health, prevention and treatment.

A recent genome-wide association study of schizophrenia (Schizophrenia-Working-Group-of-the-Psychiatric-Genomics-Consortium, 2014) identified a locus in the *CHRNA5-A3-B4* gene cluster on chromosome 15, which has been consistently shown to be associated with heaviness of smoking (Tobacco-and-Genetics-Consortium, 2010). One possible explanation for this finding is that this signal captures a *causal* effect of cigarette smoking on schizophrenia (Gage and Munafo, 2015). There is a precedent for this pattern of results: the same region was shown to be associated with lung cancer risk (Thorgeirsson et al., 2008) but it is likely that this effect arises entirely via cigarette smoking (Munafo et al., 2012).

Intriguingly, several other recent studies have been published which also support a causal role for smoking in schizophrenia and psychosis (Gurillo et al., 2015, Kendler et al., 2015, McGrath et al., 2015, Wium-Andersen et al., 2015). One study reported a stratified analysis which suggests an association of *CHRNA5-A3-B4* genotype with antipsychotic medication prescription (as a proxy of psychotic illness) in ever smokers but not in never smokers (Wium-Andersen et al., 2015). Another used Swedish registry data to show that cigarette smoking predicted subsequent diagnosis of schizophrenia, and this association was not substantially altered either by potential confounders either using standard regression methods or co-relative analyses, or by the inclusion of a buffer period to account for the possibility that prodromal symptoms of schizophrenia prior to a diagnosis might lead to the uptake of smoking (Munafo et al., 2012).

Most studies to date have focused on cigarette smoking, largely because this is by far the most common form of tobacco use. However, other forms of tobacco use exist, such as smokeless forms including oral preparation such as Swedish snuff (or “snus”). Snus is a most powder tobacco product, typically sold in prepackaged pouches and usually placed under the upper lip. In general, snus use is associated with lower levels of harm than cigarette smoking (Le Houezec et al., 2011), although the evidence with respect to psychiatric outcomes is limited. We therefore explored whether a similar pattern of association is seen between snus use and schizophrenia and non-affective psychotic illness in a large Swedish registry data set. There is clear evidence that schizophrenia lies at the end of a continuum of vulnerability to psychotic-like symptoms and psychosis. Including non-affective psychosis therefore allowed us to increase statistical power while still addressing our underlying question.

## 2 Methods

### 2.1 Participants

We linked nationwide Swedish registers via the unique 10-digit identification number assigned at birth or immigration to all Swedish residents. The identification number was replaced by a serial number to ensure anonymity. Our database contained the following sources: the Multi-Generation Register, the Swedish Hospital Discharge Register, the Swedish Prescribed Drug Register, the Outpatient Care Register, the Primary Health Care Register, the Swedish Crime Register, the Swedish Suspicion Register, the Military Conscription Register, the Population and Housing Censuses, and the Total Population Register. More information on these data sources is provided as Supplementary material. Males with valid snus and smoking assessments, aged 18–25 at the time of conscription were eligible for inclusion. During the period sampled, all Swedish males were required by law to attend two days of evaluation for conscription. Only individuals with prior disabilities or serious criminal or behavioral disturbances were exempted. Around 97% of males are included in this sample. End of follow-up was the last year of information available, which for most registries was 2010.

### 2.2 Measures

We identified smoking and snus habits in young males from the Military Conscription Register. Snus use was assessed as “Yes” or “No”, while smoking was assessed as follows: (1) “Not smoking”, (2) Light smoking—(Winkleby et al., 2007) “1–10 cigarettes/day”, or “1 packet of tobacco/week”, (3) Average smoking—“11–20 cigarettes/day”, or “1–2 packets/week”, and (4) heavy smoking—“>20 cigarettes/day”, or “>2 packets/week.” Schizophrenia (SZ) was defined in the Swedish Hospital Discharge Register by the following ICD 10 codes: F20.0, F20.1, F20.2, F20.3, F20.5, F20.8, and F20.9. Non-affective psychosis (NAP) was defined by the ICD 10 code: F2. More information on these codes is provided as Supplementary material.

Drug abuse (DA) was defined as follows: in the Swedish medical registries by ICD 10 codes: F10-F19, except (F10) or (F17); in the Suspicion Register by codes 3070, 5010, 5011, and 5012, that reflect crimes related to DA; and in the Crime Register by references to laws covering narcotics (law 1968:64, paragraph 1, point 6) and drug-related driving offences (law 1951:649, paragraph 4, subsection 2 and paragraph 4A, subsection 2). DA was identified in individuals (excluding those suffering from cancer) in the Prescribed Drug Register who had retrieved (in average) more than four defined daily doses a day for 12 months from either of Hypnotics and Sedatives (Anatomical Therapeutic Chemical (ATC) Classification System N05C and N05BA) or Opioids (ATC: N02A). These levels were set to be beyond what any responsible physician in Sweden would prescribe for anxiety or pain and would only arise from someone who is abusing the medications, often through getting multiple prescriptions from different physicians. This method of drug abuse assessment is validated by the strong correlation in probability of registration for drug abuse from medical and crime registries.

Family level socioeconomic status was assessed by low parental education, defined as elementary school only to index low educational attainment. Neighborhood level socioeconomic status was assessed by a composite measure of neighbourhood deprivation (Winkleby et al., 2007), which has been validated in prior studies (Chaikiat et al., 2012, Winkleby et al., 2007). In the year of conscription, this measure was classified into low, mid and high. This approach avoids problems associated with classifying socioeconomic status at the individual level, which may be impacted by current and recent behavior (e.g. drug use).

### 2.3 Statistical analysis

We investigated the association between snus and time to diagnosis in males not diagnosed with non-affective psychosis (including schizophrenia) before conscription with Cox proportional hazard methods, censoring at death or end of follow-up. The association between smoking and schizophrenia/non-affective psychosis is known and we therefore included the smoking × snus interaction in the model.

In addition to unadjusted analyses we adjusted for socioeconomic status, assessed as parental education at the individual level and neighbourhood deprivation, and DA before SZ/NAP onset. To facilitate interpretation, we present the snus associations separately by smoking category. Statistical analyses were performed using SAS 9.3 (18).

## 3 Results

### 3.1 Characteristics of participants

Of the 227,117 individuals fulfilling the inclusion criteria, 60,804 (26.8%) reported being snus users. The vast majority of participants (N = 223,412, 98.4%) were aged 18 or 19 at the time of assessment. A full description of the characteristics of participants, including snus users and non-users, is provided in Table 1.

### 3.2 Association of snus use with schizophrenia and non-affective psychosis

There was a positive association between snus use and odds of schizophrenia in all analyses, but the magnitude of the associations were small and the confidence intervals wide (unadjusted HR 1.13, 95% CI 0.77–1.67; partially adjusted HR 1.14, 955CI 0.77–1.68; fully adjusted HR 1.03, 95% 0.70–1.54) (Table 2). However, the small number of schizophrenia cases in these analyses meant that statistical power was low.

A similar pattern was observed for non-affective psychosis (including schizophrenia), but the magnitude of the association was somewhat greater and the confidence intervals narrower. These analyses provided statistical evidence that this association was unlikely due to chance effects (unadjusted HR 1.33, 95% CI 1.10–1.61; partially adjusted HR 1.33, 95% CI 1.09–1.61; fully adjusted HR 1.22, 95% CI, 1.00–1.48) (Table 2).

### 3.3 Association by smoking status

We examined the relationship between snus use and risk for schizophrenia and non-affective psychosis separately by cigarette smoking status (Table 2). Evidence for an association was only reliably seen in non-smokers where, for non-affective psychosis, the HR was approximately 1.4 and changed little with adjustment.

## 4 Discussion

Our results provide modest evidence for an association between snus use and risk for non-affective psychosis. Although a broadly similar pattern of results was observed for snus use and schizophrenia, our analysis was underpowered for this outcome, making it difficult to draw any conclusions with confidence. When we considered snus use separately in non-smokers and smokers we observed similar evidence for an association with non-affective psychosis but only in non-smokers, and not in smokers. While the magnitude of the associations we observed was modest, for a common exposure such as tobacco use the population level impact of any causal effect will nevertheless be substantial.

It is notable that the association between snus use and risk for non-affective psychosis was only observed in non-smokers. This may be because, among smokers who also use snus, cigarettes represent the major source of tobacco exposure. However, we also observed evidence among smokers of a modest negative correlation between snus use and cigarette use. This may reflect partial nicotine substitution among dual users, where snus use leads to a corresponding reduction in cigarette smoking, given that tobacco users typically titrate intake to achieve the desired nicotine level. Dissecting the relative contribution of snus and cigarette exposure among dual users is therefore likely to be complex. Taken together, these results provide some evidence against the hypothesis that it is the burnt products of cigarette smoke that are psychotogenic.

There are some limitations that should be considered when interpreting these results. First, despite the relatively large sample size, our study was most likely underpowered to detect modest associations between snus use and a rare outcome such as schizophrenia. However, the overall pattern of results was broadly similar for both outcomes, which increases confidence somewhat in these findings. Second, as with any observational analysis, we cannot fully exclude the possibility that residual confounding or reverse causality may account for the associations we observed. However, the current results are consistent with emerging evidence from a range of studies and methodologies that tobacco use may be a risk factor for psychotic illness. Third, the sample included only conscripted males, and was predominantly of European ancestry, so that the findings are not necessarily generalizable to other populations. It is worth noting that attending the conscript evaluation is mandatory for males in Sweden, so that the sample should be representative of the male Swedish population over the period sampled.

In conclusion, our data provide some evidence that snus use is associated with the subsequent development of non-affective psychosis. The evidence for an association with schizophrenia is weaker, but broadly consistent. Given growing evidence that tobacco use may play a role in the aetiology of psychotic illness, these results are potentially important in that they suggest that any causal agent is present in unburned tobacco. One candidate agent is nicotine, which influences dopaminergic signaling in several brain regions (Subramaniyan and Dani, 2015). However, there are other constituents of tobacco that are also plausible candidates, such as monoamine oxidase inhibitors (Hogg, 2015). If tobacco use is in fact a risk factor for psychotic illness, it will be important to identify the constituents responsible, in particular given growing interest in the use of nicotine-containing products (e.g., electronic cigarettes) for harm reduction, including among psychiatric patients.

## Conflict of interest

No conflict declared.

## Role of funding source

Nothing declared.

## Contributors

Marcus R. Munafò conceived the study, drafted the manuscript, and approved the final manuscript for submission.

Sara Larsson Lönn conducted statistical analyses, and approved the final manuscript for submission.

Jan Sundquist created the registry resources used in the manuscript, advised on the analyses and approved the final manuscript for submission.

Kristina Sundquist, created the registry resources used in the manuscript, advised on the analyses and approved the final manuscript for submission.

Kenneth Kendler conceived the study, drafted the manuscript, and approved the final manuscript for submission.

## Figures and Tables

**Table 1:** Characteristics of participants.

	All N = 227,117	Not using snus N = 166,313	Using snus N = 60,804
Birth year, mean (SD)	1986.4 (2.0)	1986.5 (2.0)	1986.2 (1.9)
Age at conscript, mean (SD)	18.2 (0.5)	18.2 (0.5)	18.3 (0.5)
Age at end of follow up, mean (SD)	26.1 (2.1)	26.1 (2.1)	26.3 (2.0)
With non-affective psychosis (%)	473 (0.21%)	316 (0.19%)	157 (0.26%)
With schizophrenia (%)	120 (0.05%)	84 (0.05%)	36 (0.06%)
Low neighbourhood deprivation (%)	57,125 (25.2%)	43,195 (26.0%)	13,930 (22.9%)
Medium neighbourhood deprivation (%)	137,047 (60.3%)	98,885 (59.5%)	38,162 (62.8%)
High neighbourhood deprivation (%)	31,941 (14.1%)	23,513 (14.1%)	8428 (13.9%)
Low parental education (%)	24,456 (10.8%)	16,840 (10.1%)	7616 (12.5%)
Drug abuse (before diagnosis) (%)	15,206 (6.7%)	8496 (5.11%)	6710 (11.0%)

**Table 2:** Association of snus use with schizophrenia/non-affective psychosis, by smoking status.

	Unadjusted	Partially adjusted^a^	Fully adjusted^b^
	Schizophrenia		
Combined	1.13 (0.77, 1.67)	1.14 (0.77, 1.68)	1.03 (0.70, 1.54)
Non-smoker	1.29 (0.81, 2.08)	1.30 (0.81, 2.08)	1.23 (0.77, 1.98)
Light smoker	0.38 (0.15, 0.97)	1.39 (0.15, 1.01)	0.42 (0.16, 1.07)
Moderate smoker	0.67 (0.17, 2.57)	0.69 (0.18, 2.70)	0.75 (0.19, 2.92)
Heavy smoker	1.22 (0.25, 6.04)	1.26 (0.26, 6.22)	1.43 (0.29, 2.08)
	Non-affective psychosis		
Combined	1.33 (1.10, 1.61)	1.33 (1.09, 1.61)	1.22 (1.00, 1.48)
Non-smoker	1.44 (1.14, 1.82)	1.45 (1.15, 1.83)	1.38 (1.09, 1.75)
Light smoker	0.65 (0.43, 0.99)	0.66 (0.43, 1.00)	0.69 (0.45, 1.05)
Moderate smoker	0.87 (0.45, 1.67)	0.90 (0.47, 1.74)	0.97 (0.50, 1.87)
Heavy smoker	0.54 (0.17, 1.75)	0.56 (0.17, 1.81)	0.63 (0.19, 2.06)

Values represent hazard ratios (95% confidence interval). Light smoking is defined as 1–10 cigarettes/day, or 1 packet of tobacco/week, moderate smoking as 11–20 cigarettes/day, or 1–2 packets/week, and heavy smoking as >20 cigarettes/day, or >2 packets/week. Analyses in combined sample are adjusted for smoking status.

a Additionally adjusted for neighbourhood deprivation, parental education.

b Additionally adjusted for drug abuse prior to diagnosis.
