# Supplementary material for: Snus use and risk of schizophrenia and non-affective psychosis
Source: Drug Alcohol Depend. 2016 Jul 1;164:179–82. doi: 10.1016/j.drugalcdep.2016.04.035 (PMC4907123; doi:10.1016/j.drugalcdep.2016.04.035)
Supplement: Supplementary file 1 [file mmc1.docx]

**Supplementary Material for the article**

Snus Use and Risk of Schizophrenia and Non-Affective Psychosis

Marcus R. Munafò ^1,2^, Sara Larsson Lönn ^3^, Jan Sundquist ^3^, Kristina Sundquist* ^3^, Kenneth Kendler* ^4^

1. MRC Integrative Epidemiology Unit at the University of Bristol, Bristol, United Kingdom.

2. UK Centre for Tobacco and Alcohol Studies, School of Experimental Psychology, University of Bristol, Bristol, United Kingdom.

3. Center for Primary Health Care Research, Lund University, Malmö, Sweden

4. Virginia Institute for Psychiatric and Behavioral Genetics, Department of Psychiatry, Virginia Commonwealth University School of Medicine, Richmond, VA, USA.

* These authors jointly supervised this work.

Corresponding author: Marcus Munafò, School of Experimental Psychology, University of Bristol, 12a Priory Road, Bristol BS8 1TU, United Kingdom. E: marcus.munafo@bristol.ac.uk

**This material supplements but does not replace, the peer-reviewed article in *Drug and Alcohol Dependence*.**

The following sources contributed to the study database: the Multi-Generation Register, providing information on family relationships for individuals born in Sweden after 1932 and corresponding information for immigrants who became citizens of Sweden before age 18 together with one or both parents; the Swedish Hospital Discharge Register, containing hospitalizations for Swedish inhabitants from 1964-2010; the Swedish Prescribed Drug Register, containing all prescriptions in Sweden collected by patients from 2005 to 2009; the Outpatient Care Register, containing information from all outpatient clinics from 2001 to 2010; the Primary Health Care Register, containing outpatient primary care data on diagnoses from 2001 to 2007 for 1 million patients from Stockholm and parts of southern Sweden; the Swedish Crime Register, including all convictions in lower court from 1973 to 2011; the Swedish Suspicion Register, including all individuals strongly suspected of crime from 1998 to 2011; the Military Conscription Register, including smoking and snus habits from 2002 to 2008; the Population and Housing Censuses, including information on household and geographical status in 1960, 1965, 1970, 1975, 1980, and 1985; and the Total Population Register, containing annual data on family and geographical status in 1990-2010. Males with valid snus and smoking assessments, aged 18 to 25 at the time of conscription were eligible for inclusion.

The F2 code in ICD 10 is termed “Schizophrenia, schizotypal and delusional disorders” and includes the following main categories: F20 – schizophrenia, F21 – schizotypal disorders, F22 and F24 - persistent delusional disorders, F23 - acute and transient psychotic disorders – F25 - schizoaffective disorders, F28 - other nonorganic psychotic disorders, and F29 - unspecified nonorganic psychosis. So schizoaffective disorder was included in our category of nonaffective psychosis, consistent with the following ICD 10 definition: “Episodic disorders in which both affective and schizophrenic symptoms are prominent but which do not justify a diagnosis of either schizophrenia or depressive or manic episodes.”
